# Transformation of Graphite Recovered from Batteries into Functionalized Graphene-Based Sorbents and Application to Gas Desulfurization

**DOI:** 10.3390/molecules29153577

**Published:** 2024-07-29

**Authors:** Rodolfo Fernández-Martínez, Isabel Ortiz, M. Belén Gómez-Mancebo, Lorena Alcaraz, Manuel Fernández, Félix A. López, Isabel Rucandio, José María Sánchez-Hervás

**Affiliations:** 1 Chemistry Division, Technology Department, Research Centre for Energy, Environment and Technology (CIEMAT), 28040 Madrid, Spain; mariabelen.gomez@ciemat.es (M.B.G.-M.); m.fernandez@ciemat.es (M.F.); isabel.rucandio@ciemat.es (I.R.); 2 Sustainable Thermochemical Valorization Unit, Energy Department, Research Centre for Energy, Environment and Technology (CIEMAT), 28040 Madrid, Spain; isabel.ortiz@ciemat.es (I.O.); josemaria.sanchez@ciemat.es (J.M.S.-H.); 3 National Centre for Metallurgical Research (CENIM), Spanish National Research Council (CSIC), Avda. Gregorio del Amo 8, 28040 Madrid, Spain; alcaraz@cenim.csic.es (L.A.); f.lopez@csic.es (F.A.L.)

**Keywords:** Zn/C battery waste, reduced graphene oxide, rGO-based composites, desulfurization, syngas cleaning, sustainability

## Abstract

The recycling and recovery of value-added secondary raw materials such as spent Zn/C batteries is crucial to reduce the environmental impact of wastes and to achieve cost-effective and sustainable processing technologies. The aim of this work is to fabricate reduced graphene oxide (rGO)-based sorbents with a desulfurization capability using recycled graphite from spent Zn/C batteries as raw material. Recycled graphite was obtained from a black mass recovered from the dismantling of spent batteries by a hydrometallurgical process. Graphene oxide (GO) obtained by the Tour’s method was comparable to that obtained from pure graphite. rGO-based sorbents were prepared by doping obtained GO with NiO and ZnO precursors by a hydrothermal route with a final annealing step. Recycled graphite along with the obtained GO, intermediate (rGO-NiO-ZnO) and final composites (rGO-NiO-ZnO-400) were characterized by Wavelength Dispersive X-ray Fluorescence (WDXRF) and X-ray diffraction (XRD) that corroborated the removal of metal impurities from the starting material as well as the presence of NiO- and ZnO-doped reduced graphene oxide. The performance of the prepared composites was evaluated by sulfidation tests under different conditions. The results revealed that the proposed rGO-NiO-ZnO composite present a desulfurization capability similar to that of commercial sorbents which constitutes a competitive alternative to syngas cleaning.

## 1. Introduction

The global production and demand for graphite has increased in recent years, largely because of the use of graphite for producing batteries of electric vehicles. In 2022, the global consumption of graphite reached 3.8 million tons, compared to 3.6 million tons in 2021 [1]. Actually, mining continues to be the main source of graphite with 1.6 million tons in 2023 [2]. It is essential to preserve carbonaceous natural resources by the application of sustainable approaches for graphite production whilst reducing the environmental impact. According to the principles of a circular economy, recycling graphite from devices where it is present may contribute to the key challenge for achieving the objective of closed-loop system materials and sustainability [3].

Cathodes from Zn/C batteries are made of graphite carbon. The recycling process is based on a hydrometallurgical process in a preliminary step of the dismantling of batteries followed by acidic leaching [4]. This yields a black mass consisting of a mixture of graphite and metallic oxides. In spite of the presence of metallic impurities, recovered graphite can be used as a precursor to prepare high-added-value products such as graphene-related materials and graphene-based composites [5,6,7].

Graphene is a 2D material that has recently received an enormous amount of attention due to their exceptional physicochemical properties. It is constituted of a single-atom-thick layer of conjugated sp2 carbon atoms arranged in a honeycomb structure. The top-down approach based on chemical exfoliation constitutes the most standardized method to synthesize graphene-related materials, such as graphene oxide (GO) or reduced graphene oxide (rGO) [8,9]. Among its strengths is its versatility, scalability and tunability in terms of the surface area, size and functionality while its weaknesses lie in the presence of defects and remaining functional groups due to the incomplete reduction of oxygen groups [10]. To date, chemical exfoliation represents the most suitable route to produce large amounts of graphene at a reasonable cost from graphite oxide [11]. Graphene oxide (GO) is synthesized from graphite under strong oxidative conditions and subsequent exfoliation. Afterwards, GO may be converted to reduced graphene oxide (rGO) by a reduction process where the oxygen-containing groups are partially removed. 

rGO presents characteristics that make it an ideal support to prepare hybrid sorbents. Among them, the most crucial in order to prepare rGO-based composites are the high surface-to-volume ratio, owing to its 2D nature, its porous structure as well as its remaining oxygenated groups, which act as anchoring sites to active groups, and the presence of vacancies that serve as a trap for incoming atoms and nanoclusters [12]. This allows the possibility of incorporating a wide variety of groups that determine the functionality of the resulting composites. Potential applications of rGO-based sorbents include the retention and removal of pollutants and energy storage [13,14,15,16,17]. In a recent study, the suitability of rGO-NiO-ZnO-based sorbents for hydrogen sulfide removal from syngas has been demonstrated [18].

Gasification is a thermochemical process that under sub-stoichiometric oxygen converts biomass or waste into a gaseous chemical energy carrier, known as synthesis gas, which can be used to produce bioenergy or further transformed into a variety of gaseous and liquids fuels such as hydrogen, synthetic natural gas (SNG), jet fuels, or chemicals, e.g., methanol or dimethyl ether (DME) [19]. 

One issue of concern is the presence of sulfur species in the raw syngas since all the above transformation processes rely on the use of catalysts, which are extremely sensitive to deactivation by sulfur compounds. Zinc oxide-based sorbents have been demonstrated to achieve gas desulfurization targets. In addition to that, sulfur removal with such sorbents can be nicely coupled with thermochemical biofuel catalytic production given that they work in similar temperature windows. Their main drawback is their cost. There is a clear need for the development of cheaper materials and their synthesis from recovered materials is a good way [20]. 

In the recent literature, there have been several works based on the use of recycled graphite from spent Zn/C batteries to synthesize graphene-related materials [21]. Composite materials have been used as a support to energy applications such as supercapacitors [22] or to fuel hydrogen production [6]. However, to the best of our knowledge, no prior studies have considered the preparation of rGO-based sorbent for the removal of sulfur pollutants from renewable fuels produced by catalytic processes. 

In this work, the synthesis of cost-effective and efficient rGO-based sorbents with a gas desulfurization capability is proposed. The black mass recovered from exhausted Zn/C battery rods is leveraged as a low-cost and sustainable graphite source to be used as a precursor of graphene oxides. Previous studies from Zn/C batteries are typically based on the use of strong leaching conditions that generate waste with difficult management [5]. In the proposed work, a previously assessed process to recovery the metals from Zn/C batteries, allowing the production of Zn/Mn oxides with varying stoichiometry for various applications [23], has been performed. This method results in a carbonaceous insoluble residue, which is suitable for its use as a raw material to obtain carbon-based materials. Furthermore, the use of this solid residue allows the achievement of a fully sustainable zero-waste process. Further functionalization with Ni and Zn reagents allows the obtainment of rGO-NiO-ZnO composites. The developed composites have been evaluated as sorbents to remove hydrogen sulfide and compared to other commercially available sorbents. rGO-based sorbents from recycled graphite may contribute to the circular economy by the valorization of wastes that are typically scrapped.

## 2. Results

Starting from graphite previously expanded by treatment with sulfuric acid and hydrogen peroxide, functionalized reduced graphene oxides have been synthesized, as described in Section 3.3. The synthesized materials as well as the starting materials have been chemically and structurally characterized to confirm the presence, in each case, of the desired material with both the catalytic activity (ZnO and NiO) and support capacity (rGO). 

The synthesized materials have been named as set out in Table 1 below:

### 2.1. Chemical Characterization of the Materials

All the materials were analyzed using the X-ray fluorescence (XRF) technique, which allows the direct analysis of the solid. The analysis was carried out using helium gas as a medium and introducing the samples directly into a specific sample holder for powder samples. 

The elemental characterization performed by the Wavelength Dispersive X-ray Fluorescence (WDXRF) technique is able to determine the elements present in the sample between oxygen and uranium and in concentrations ranging from a percentage (%) to parts per million (mg/kg). The semi-quantitative method used in the analysis, developed by Malvern Panalytical, allows the rapid analysis of the elements in the sample. The results of the analyses carried out on the materials involved in this work are shown in Table 2, expressed as a %.

All samples show moderate-to-high C concentrations (Table 2). The GRERV21-MX-SA sample, recycled graphite, shows the highest percentage (63%). As the oxygenated groups have been introduced between the graphite sheets upon oxidation, to obtain the graphite oxide, GO-17 sample, as expected, the concentration of carbon in the GO samples decreases to around 40%. These C concentrations are significantly lower than those observed in GO samples obtained by the Tour method from high-purity natural graphite [24,25]. This means that the oxidation process takes place in a higher extent resulting in a more oxidized product with a greater abundance of oxygen groups. In the functionalized reduced-graphene oxide material, the rGO-NiO-ZnO sample, the C concentration has been considerably reduced, as expected [26]. This result corroborates the tendency of rGO-NiO-ZnO to recover the original graphite structure. On the other hand, high concentrations of the elements Ni and Zn are observed, corresponding to 5 and 37%, respectively, demonstrating the successful introduction of Ni and Zn into the structure of the rGO material. These two metal oxides are the active species for hydrogen sulfide removal at an intermediate temperature. In the thermal reductions of graphene oxides, a violent process of CO and CO_2_ emission occurs [27] which causes part of the oxygen along with part of the C to be removed as a gas. This effect along with the introduction of a large proportion of Zn and Ni phases produces an important reduction of the C concentration in the rGO-NiO-ZnO-400 sample. The removal of carbon and oxygen in the form of CO and CO_2_ results in a pre-concentration of the elements Ni and Zn, whose concentrations correspond to 6.2 and 47%, respectively, which are comparable to those observed for composites synthesized from natural graphite in the same experimental conditions [18]. However, the C percentage in the composite (rGO-NiO-ZnO-400) is significantly lower than that obtained from natural graphite (3.9% vs. 11.0%). The reason might be the presence of remaining unreduced oxygen groups probably because of the great abundance of oxygen groups in the GO which results in a lesser degree of reduction. 

The results presented in Table 2 show that in the GRERV21-MX-SA sample, the most abundant element after C is Mn (9.6%), an element coming from the cathode of Zn/C batteries. A high concentration of Ba is also observed, corresponding to 1.3%. However, in the GO-17 sample, all the elements, with the exception of C, are in a low concentration, below 2%. The most abundant element after C is found to be S, which may be partly due to the oxidation process. Ba, with a concentration of 1.2% in the GO-17 sample, presents a concentration equivalent to that in the starting graphite (GRERV21-MX-SA). The rest of the elements, except C, Ni and Zn, appearing in the rGO samples are in a very low concentration, including Ba. This means that the oxidation process applied to the sample is able to remove the major elements present in the original sample with the exception of barium. The concentration of this element strongly decreases in the rGO samples as a consequence of thermal annealing.

### 2.2. Structural and Textural Characterization of the Materials

To determine the crystalline structures present in the materials, X-ray diffraction (XRD) was performed. Table 3 shows the crystalline phases found in the synthesized materials.

**Figure 1:**
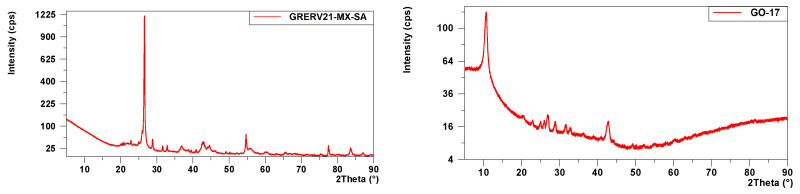

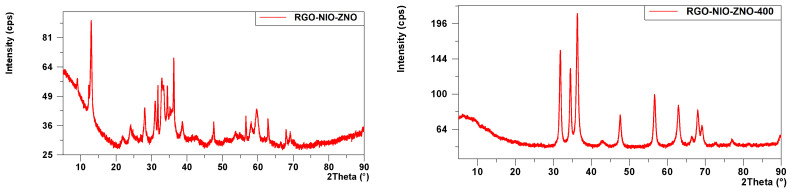
XRD patterns for the synthesized materials.

As can be seen in Table 3, the starting sample (GRERV21-MX-SA) is mainly composed of graphite (Figure 1), with a main peak around 26° (2Ɵ). BaSO_4_, a rather insoluble compound, and SiO_2_ phases are also identified. Figure 1 shows that in the GO-17 sample, a main peak can be seen at approximately 10° (2Ɵ) corresponding to graphene oxide [28]. This result corroborates the complete transformation of graphite to graphene oxide, with the disappearance of the peak around 26° (2Ɵ). This peak is quite broad, with a width at mid-height corresponding to 1.71° (2θ) and a low intensity, which indicates that adequate oxidation and exfoliation of the graphite has occurred [28], corroborating the data obtained by the WDXRF technique. In addition, a BaK_x_SO_4_ phase similar to the one found in the GRERV21-MX-SA sample appears, since it has not been possible to remove the Ba with the treatment employed, as it corresponds to a rather insoluble compound, which may come from the starting graphite.

The functionalization and reduction process has resulted in the sample labeled rGO-NiO-ZnO. This sample also shows a poorly crystalline profile with low intensity peaks (Figure 1). Furthermore, a very broad band between 20 and 30° (2Ɵ) appears, corresponding to the reduced graphene oxide (rGO) [27]. 

The crystalline phases found in rGO-NiO-ZnO correspond, in their totality, to compounds with Zn (Table 3); however, from Table 2 it can be deduced that also 5% Ni has been introduced in the sample during the hydrothermal treatment. Ni when is introduced into graphene structures gives rise to amorphous structures (probably nickel oxyhydroxides), which could be anchored via covalent bonds to the graphene sheets [26]. The presence of these partially oxidized phases is typically from composites derived from hydrothermal routes and it has been previously observed in similar structures [18,29]. The crystalline Zn compounds that have been identified in this sample (Table 3) correspond to zinc oxide as well as other compounds such as oxyhydroxides formed during the hydrothermal process [30] that are the ultimate precursors of zinc oxide.

The analysis of the rGO-NiO-ZnO-400 material is shown in Figure 1. The profile of this material is more crystalline with more defined peaks. The phases found (Table 3) correspond to Zn (ZnO) and Ni (Ni_0.7_Zn_0.3_O) oxides, although the latter is doped with a small proportion of Zn. This corroborates the results obtained in the WDXRF analysis and demonstrates the effectiveness of the annealing treatment at 400 °C to effectively complete the oxidation of oxyhydroxide’s intermediate phases into ZnO and NiO.

Textural characterization was carried out by assessing the specific surface area (SSA), applying the physical adsorption of nitrogen (N2) at 196 °C, and the results were calculated according to the Brunauer–Emmett–Teller (BET) method. Also, the pore volume was calculated by applying t-plot methodology.

This textural characterization has been applied on the rGO-NiO-ZnO-400 sample as this is the sample used in the proposed application. This sample has a specific surface area (SSA) of 46.48 m^2^·g^−1^ and t-Plot micropore volume of 0.003385 cm^3^·g^−1^, which is similar to that obtained with the same treatment on pure graphite [18]. In a previous work, J. M. Sánchez-Hervas et al. synthesized several materials similar to ZnO-NiO-400 from pure graphite. In particular, the so-called rGO (5) (Zn-Ni-rGO) had the same synthesis as rGO-ZnO-NiO-400. In this case, the SSA corresponded to 46.42 m^2^·g^−1^ and a t-Plot micropore volume of 0.0028 cm^3^·g^−1^. 

The BET and t-Plot measurements show the rGO specific surface area and micropore volume data, which are comparable to those measured in rGO obtained from non-recycled graphite precursors.

### 2.3. Sulfidation Tests

The reduced graphene rGO-NiO-ZnO-400 sample has been studied as a desulfurization sorbent. 

The H_2_S removal ability of the same type of sorbents synthetized from pure graphite was demonstrated in a previous study [18]. The reactive adsorption of H_2_S on rGO/metal hydroxides occurs via acid–base reactions. Through this mechanism, H_2_S is effectively retained on the surface of the adsorbent by the direct replacement of OH groups and the acid–base reaction with the metal (hydr)oxides, resulting in the formation of sulfites and sulfates. In this work, the performance of the rGO-NiO-ZnO-400 sorbent in three different atmospheres is presented. Firstly, a simplified atmosphere with 0.9% (*v*/*v*) in nitrogen is used to compare the performance of this sorbent with the ones studied in our previous research. Then, two different syngas compositions representative of biomass gasification processes were employed.

To compare the performance of the new sorbent with those previously evaluated, the same conditions were studied, namely: 400 °C of temperature, 10 bar of pressure and a gas space velocity of 3500 h^−1^ with a gas stream containing 9000 ppmv of H_2_S/N_2_.

The gas’ hourly space velocity, GHSV, is the ratio of the volumetric gas-flow-rate in normal conditions to the bulk sorbent volume loaded into the reactor. The selected values for the desulfurization operating conditions were set in accordance with previous studies published by the authors [31].

The performance of the sorbents has been evaluated by the S loading capacity and actual breakthrough times compared to the theoretical values. To determine these theoretical values, two sulfidation reactions were considered:


ZnO +H_2_S → ZnS + H_2_O (1)

NiO +H_2_S → NiS + H_2_O (2)

The theoretical S load capacity (S_0_) is, therefore, calculated following the equation:

(3) $$S_{0}\left(\%\right)=\left(\frac{{\%}_{ZnO}}{{MW}_{ZnO}}+\frac{{\%}_{NiO}}{{MW}_{NiO}}\right)\cdot {MW}_{S}$$

And the theoretical sorption time, t_0_, when complete sulfidation is achieved, was calculated as the ratio between the theoretical amounts of S that each material can adsorb (g) based on its composition and the S mass flow rate used in each experiment (g/min). This procedure assumes that S is totally retained by the sorbent and no S escapes in the gas outlet.

(4) $$t_{0}\left(min\right)=\left(\frac{S_{0}\left(\%\right)\cdot Msorb\;(g)}{{MW}_{S}\;\cdot \;\frac{P\;\cdot Q_{H2S}}{R\;\cdot T}}\right)$$

where *Msorb* is the mass of sorbent used for desulfurization, MW is the molecular weight of S, *P* is the absolute pressure in sulfidation conditions, *Q_H_*_2*S*_ is the volumetric gas flow rate of H_2_S in the process conditions, *R* is the universal gas constant and *T* is the absolute temperature in sulfidation conditions. The sulfidation breakthrough point was set at 0.01% (*v*/*v*).

**Figure 2:**
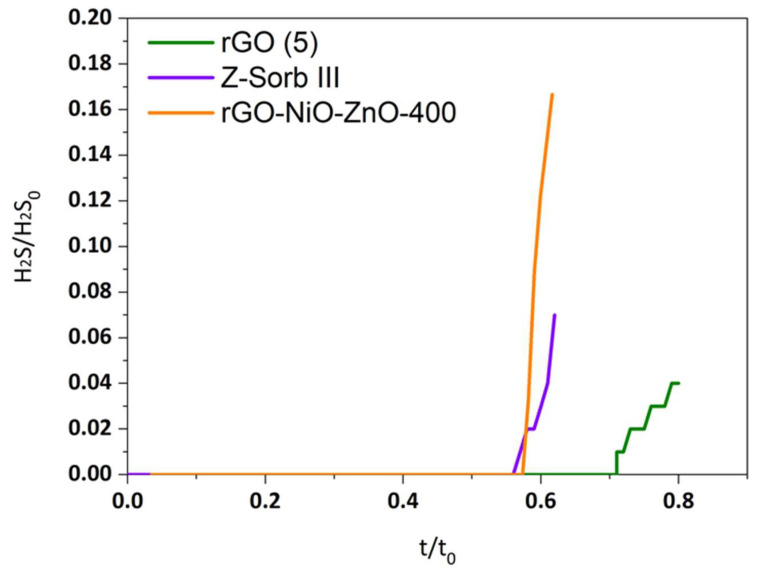
Dimensionless sulfidation breakthrough curves.

**Figure 3:**
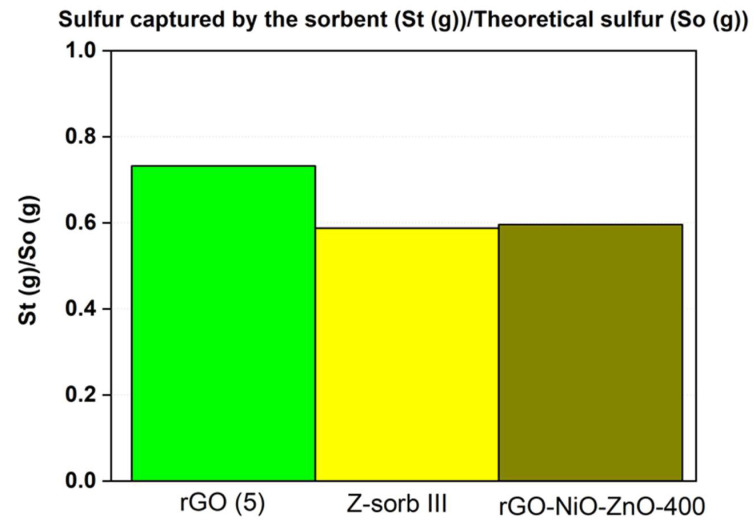
Comparison of the sorbents in terms of utilization yield.

Dimensionless breakthrough curves and the utilization yield for two sample sorbents obtained from pure graphite (rGO(5)) and from recycled graphite as well as commercial sorbents (Z-Sorb IIITM) are depicted in Figure 2 and Figure 3. As can be observed, the sorbent from recycled graphite shows a lower desulfurization capacity than the sorbents obtained from pure graphite at the same experimental conditions. This is expected since the lesser reduction observed in rGO from recycled graphite with respect to the same rGO obtained from pure graphite should result in a lower degree of recovery of the characteristic π-conjugated structure and, therefore, a lower electron mobility [32,33]. However, the desulfurization capacity remains at the levels of the commercial sorbent, Z-Sorb IIITM, indicating the suitability of the proposed sorbents for desulfurization applications.

As many gasifiers operate at atmospheric pressure and because of the difficulties of compressing a dirty syngas, the performance of the rGO-NiO-ZnO-400 sorbent was evaluated at 1 bar in a simplified atmosphere with 0.9% (*v*/*v*) in nitrogen.

**Figure 4:**
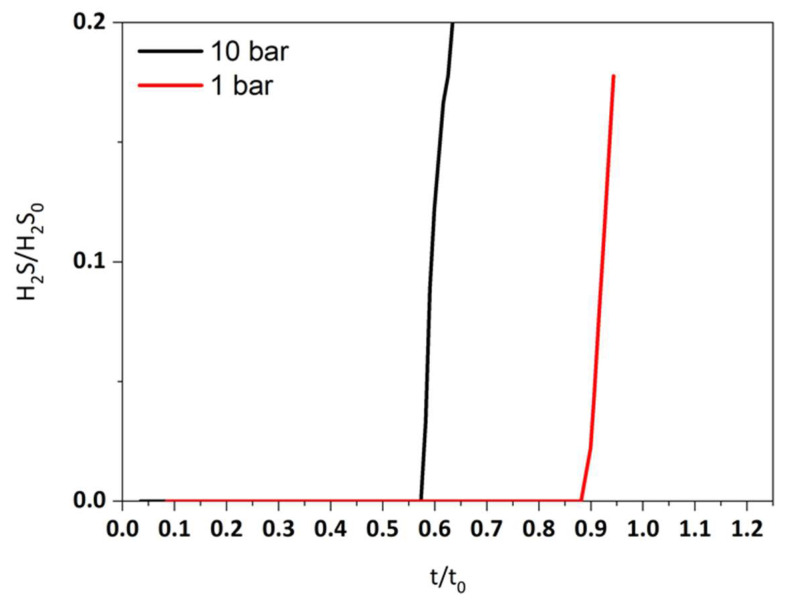
Dimensionless sulfidation breakthrough curves at different pressures.

The breakthrough curves for both operating pressures are shown in Figure 4. A closer value to the theoretical time is achieved at atmospheric pressure. Unlike the commercial sorbent which exhibits a better performance at high pressure values (2MPa) [34], the desulfurization capacity of the rGO-NiO-ZnO sorbent increases from 17.8% to 24.1% when the pressure decreases from 10 bar to 1 bar. This result is clearly advantageous since it means that in the case of the commercial application of this technology, there would be no need for gas compression upstream to the desulfurization reactor.

Since better results were obtained when a lower pressure was applied, it was decided to study the sorbent performance with synthetic syngas mixtures at 1 bar. The operating conditions, including the atmosphere composition, of the three different types of experiment carried out to determine the desulfurization capacity of the sample are summarized in Table 4.

Table 5 summarizes the results obtained. The actual S loading capacity and the breakthrough times determined in the experiment (in test 2) were close to the theoretical ones. Therefore, the utilization of the sorbent at a breakthrough time provides a very high value (efficiency).

Regarding the syngas composition, some components of the gas can interfere with sorbent sulfidation. Many examples of CO_2_ interference can be found in the literature [35,36,37,38,39] as well as CO, CH_4_ and H_2_O [35,40,41,42,43]. In this work, different composition of CO, CO_2_ and CH_4_ were used while the water content was kept constant. 

By analyzing the effect of the gas atmosphere, no significant differences were observed between the sorbent performances in tests under nitrogen and syngas atmospheres. Under a full syngas atmosphere, the sorbent did not lose its S retention capacity very significantly, which means that there is no strong competitive adsorption of or deactivation by any of the syngas components and, therefore, it does not interfere in the desulfurization process.

The mixture with a low quantity of CO and CO_2_ and higher CH_4_ (test N°2: 13.6%, 7.4% and 2.8%, respectively) exhibited a good sorbent performance. However, when a syngas composition with a higher content of CO and CO_2_ and lower CH_4_ (test N°3: 24.9%, 8.5% and 0.6%) was used, a slight decrease in the S loading capacity was observed. The S loading capacity decreased from 27% to 23%. This can be attributed to the reducing power of the gas for the second mixture which was a little bit higher. The reducing power is expressed as the ratio of reducing compounds in the syngas (sum of H_2_ + CO + CH_4_) to oxidized compounds (CO_2_ + H_2_O). For the syngas mixture, denoted as number 2, the reducing power is 2.44, whereas for the syngas mixture, number 3, it is 2.6. Moreover, in a previous study [34], the authors also observed that for rich CO syngas, the Boudouard reaction, CO disproportionation, occurred under a specific gas velocity, leading to a poor desulfurization performance. Coking due to methane cracking would also decrease the S removal capacity due to hindering access to zinc oxide and nickel oxide desulfurization sites.

**Figure 5:**
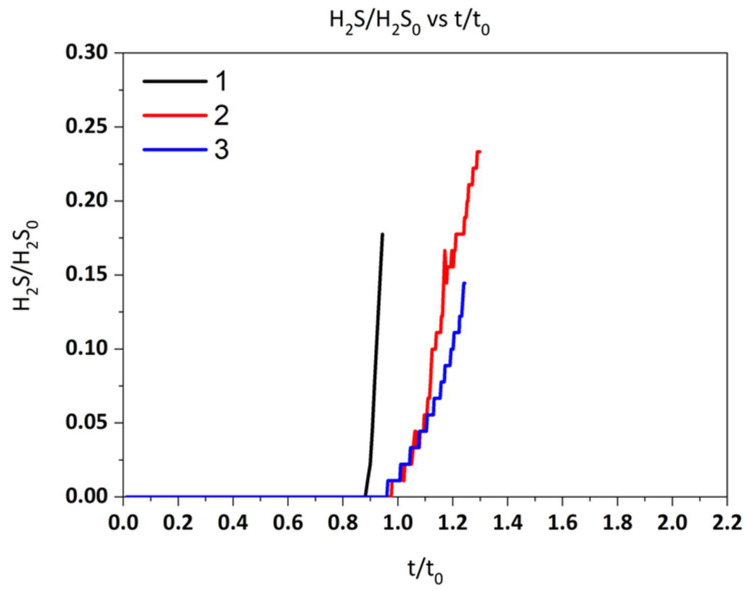
Breakthrough curves of sorbent at different conditions.

Figure 5 shows the sorbent’s dimensionless breakthrough curves of rGO-NiO-ZnO-400 for the three gas mixtures. As can be seen, there is almost no H_2_S in the exit stream prior to the breakthrough point, which is then followed by a sharp increase in the hydrogen sulfide concentration in the reactor outlet.

### 2.4. Regeneration and Ciclability of Sorbents

The capability of the sorbent was tested under the three atmospheres evaluated in order to have a preliminary insight on its performance over repeated sulfidation and regeneration cycles. To that aim, first, in a nitrogen atmosphere, three sulfidation and regeneration cycles were performed. Regeneration was carried out using a mixture of nitrogen with 2% of oxygen at 550 °C. These conditions were maintained until no SO_2_ was detected in the outlet stream and then another subsequent sulfidation test was conducted. Then, the same number of cycles was undertaken for the syngas mixture atmospheres.

**Figure 6:**
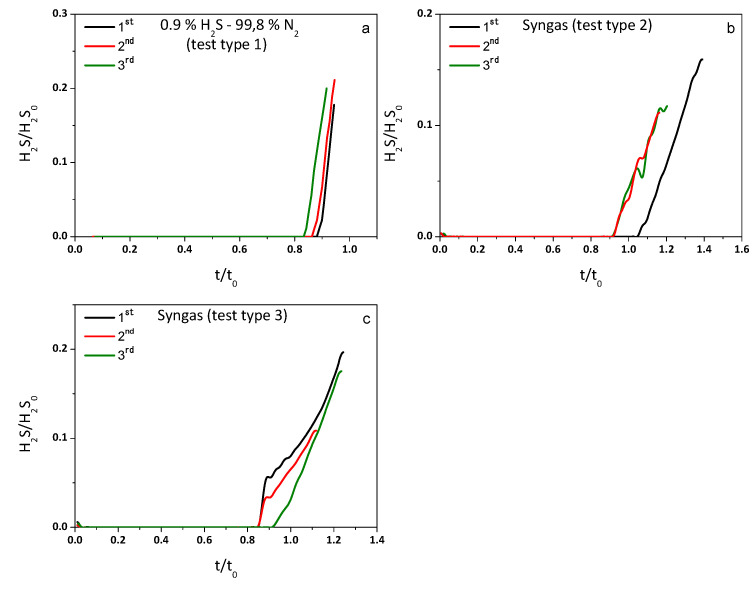
Cyclability of sorbent, (**a**–**c**) present breakthrough curves.

In Figure 6a–c, breakthrough curves are presented. A slow decrease in the sulfur retention capacity can be observed when nitrogen and the mixture with a low quantity of CO and CO_2_ and higher CH_4_ (13.6%, 7.4% and 2.8%, respectively) are used. On the other hand, when the mixture with a higher CO and CO_2_ and lower CH_4_ content (24.9%, 8.5% and 0.6%) was used, no significant difference was observed in the sorbent’s performance. Despite this negligible reduction in the sulfur retention capacity for the first syngas mixture, the efficiency of the sorbent remained high enough (see Table 6) to allow its use in several cycles.

## 3. Materials and Methods

### 3.1. Chemicals

All reagents used for graphene oxide and rGO-NiO-ZnO synthesis were of analytical reagent grade, mostly supplied by Fisher Scientific (Hampton, NH, USA). Ultrapure water (resistivity ≥ 18.2 MΩ·cm) from a Milli-Q system (Millipore Bedford, Bedford, MA, USA) was used throughout.

### 3.2. Apparatus and Instruments

Equipment used for the synthesis of graphene oxide and rGO-NiO-ZnO composites includes a thermostatic bath with heating control (Huber KISS225B, Offenburg, Germany), a rod stirrer (Selecta SE-100, Barcelona, Spain), a vacuum freeze-dryer (LaboGene Coolsafe Touch 1110-4, Allerød, Denmark), a high-capacity floor centrifuge (Gyrozen 1736R, Daejeon, Republic of Korea), an ultrasonic probe (Fisherbrand model 505, Pittsburgh, PA, USA), an automatic mill (Bosch TSM6A011W, Stuttgart, Germany), a magnetic stirrer (Selecta Multimatic 9N, Barcelona Spain), 250 mL PTFE lined autoclave reactors, a vacuum drying oven (WITEG WOV 70, Wertheim, Germany) and a gradient tube furnace (CARBOLITE TZF 1200 °C, Sheffield, United Kingdom).

X-ray diffraction (XRD) measurements were carried out using a X’Pert Pro (Malvern-Panalytical, Almelo, Netherlands) using Cu Kα radiation (λ = 1.54056 Å). The instrument was configured with Bragg–Brentano geometry and with the operating parameters of 40 kV and 45 mA. Diffractograms were acquired in the range 5–120° 2θ, with scanning steps of 0.02° 2θ. 

Phase composition and structural analyses on the materials were obtained using HighScore Plus 4.8 software (Malvern-Panalytical). Compound identification was carried out using information available from the Crystallography Open Database (COD) and the International Center for Diffraction Data (ICDD).

Chemical characterization (% and mg/kg concentrations) of all samples involved in this study was performed employing wavelength dispersive X-ray fluorescence (XRF) instrumentation by using an automated AXIOS Malvern-PANalytical spectrometer with a Rh tube.

To assess the C content of the samples, an Elemental analyzer, LECO TruSpec CHN elemental analyzer (St. Joseph, MI, USA, was employed, which was then heated up to at least 900 °C in the presence of oxygen gas. Mineral and organic compounds were oxidized and/or volatilized to carbon dioxide, which was measured by an infrared detection method.

An ASAP 2020 analyzer (Micromeritics, Norcross, GA, USA) was employed to evaluate the specific surface area and pore volume of graphene material.

### 3.3. Recovery and Treatment of Graphite from Spent Zn/C Batteries

The recycled graphite used as starting material was obtained following a procedure described below. The recovered black mass from spent Zn/C batteries was subjected to acidic leaching using a mixture of 250 mL of milliQ water, 250 mL of H_2_O_2_ and 500 mL of HCl at room temperature for 1 h [44]. After that, the final mixture was filtered using a pressure filter obtaining the carbonaceous material which will be used as a precursor. In order to remove the possible small quantities of metals that may be present, the non-soluble residue was subjected to acidic leaching using a sulfuric acid 2 M concentration solution at 80 °C. Finally, the mixture was filtered and the obtained solid was dried to achieve the corresponding recycled graphite material precursor.

### 3.4. Synthesis Methods

#### 3.4.1. Synthesis of Graphene Oxide

Graphene oxide was synthesized following the guidelines provided in the Marcano–Tour method [45] with slight modifications in order to manage the highly reactive graphite. Briefly, 3 g of recycled graphite were weighed and a 9:1 mixture of concentrated H_2_SO_4_ and H_3_PO_4_ (360:40 mL) was added. In order to avoid an explosive reaction because of the presence of remaining trace metal from the original black mass, the mixture was cooled onto crushed ice and then 18 g of KMnO_4_ was slowly added in small portions, usually four portions. The mixture was then heated to 50 °C using a temperature-controlled water bath and stirred for 18 hours, turning out into a dark purple paste. Afterwards, the content was cooled down to room temperature by adding 400 mL of ultrapure ice-water to the mixture to stop the oxidation process. Finally, 10 mL of 30 wt.% H2O2 was added in order to reduce residual KMnO_4_ to soluble MnSO_4_ in an acidic medium, as described in the following reaction:


2 KMnO_4_ + 5 H_2_O_2_ + 3 H_2_SO_4_ → 2 MnSO_4_ + K_2_SO_4_ + H_2_O + 5 O_2_ (5)

After H_2_O_2_ addition, bubbling occurred and suspension turned dark yellow, which is indicative of a high oxidation level. The obtained yellow–brown suspension was then cooled down to room temperature overnight. After its transfer to two 400 mL centrifuge tubes, the suspension was centrifuged at 8000 rpm for 1h and the supernatants removed. The obtained graphite oxide was washed repeatedly with 250 mL 1M HCl and ultrapure water until the pH of the supernatant achieved 3.5–4. The final sample was placed in a Petri dish to be deep-frozen at −80 °C for 48 h and subsequently freeze-dried under high vacuum. Finally, the obtained graphite oxide was ground using an automatic mill.

#### 3.4.2. Synthesis of rGO-NiO-ZnO Composites

rGO-NiO-ZnO composites were prepared according to experiment 5 from Sanchez-Hervas et al. [18] with several modifications in order to scale up the production. In spite of not presenting the highest adsorption capacity, the synthesis conditions were selected as a compromising solution between a high surface area, chemical stability and easiness to scalability. In a typical synthesis, 1 L of 5 mg/mL homogeneous aqueous dispersion of graphite oxide was prepared from four batches of 250 mL. Then, each 250 mL dispersion was successively sonicated in a low-power sonication bath for 1 h and with probe sonication for 3 h, producing clear graphene oxide (GO) dispersions with no visible particulate matter. After sonication process was complete, the four dispersions were joined and equally distributed in five beakers and subjected to magnetic stirring. Corresponding quantities of Zn(NO_3_)_2_, Ni(NO_3_)_2_ and urea were successively added in small portions across 3 h to the GO dispersions while stirring. The mass ratio of Zn(NO_3_)_2_:GO is 12.4:1 while the mass ratio of Ni(NO3)2:GO is 2:1. The mixture was stirred for 2 more hours. The solutions of GO and metal oxide precursors were then transferred to 250 mL PTFE-lined stainless steel autoclaves and subjected to hydrothermal treatment at 120 °C for 48 h. The obtained greyish precipitates were washed successively two times with ethanol and two times with ultrapure water and dried at 60 °C under vacuum. Finally, a thermal annealing treatment at 400 °C under Ar atmosphere was applied to obtain the NiO-ZnO-decorated rGO using a gradient tube furnace. 

### 3.5. Desulfurization Test

#### 3.5.1. Test Rig

Desulfurization tests were carried out in a Microactivity Pro Unit. A full description of the system can be found elsewhere [46]. The unit can work at up to 700 °C and 20 bar with a maximum operating gas flow rate of 4.5 NL/min. Three mass flow controllers and a HPLC pump (Gilson 307) produce the desired gas mixture composition. Dry gas and water are preheated separately up to 200 °C in two independent loops, in which water was vaporized and then mixed before entering the reactor. The sulfur-resistant tubular reactor of 9.2 mm OD and 30 cm long was placed in one single-zone SS304 oven to heat up the full gas stream to the operating temperature and controlled by a 1.5 mm thermocouple.

For the tests presented in this work, three different atmospheres were employed: one simplified atmosphere with H_2_S and nitrogen and two resembling different gasification gases. The simplified atmosphere consisted of a mixture of hydrogen sulfide (9000 ppmv) in nitrogen and no water was fed to the system during this experiment. The gasification gas composition selected was (1) 5% CH_4_, 24% CO, 13% CO_2_, 46% H_2_, 12% N_2,_ (2) 1% CH_4_, 44% CO, 15% CO_2_ and 40% H_2_. For those experiments, the dry stream mimicking the gasification gas was mixed with a stream of H_2_S in nitrogen to produce a final concentration of H_2_S of 3000 ppmv. Gas humidity was set at 10% that which was achieved by the liquid feeding system which feeds water, vaporizes it and mixes it with the dry gas stream before reaching the reactor.

Gas stream compositions were measured after water removal by gas chromatography using a CP4900 Varian gas microchromatograph equipped with two columns, a Porapack HP-PLOT Q and a Molecular Sieve HP-PLOT and with two thermal conductivity detectors. 

#### 3.5.2. Experimental Methodology

rGO-NiO-ZnO-400 composites prepared as powder were pelletized, weighed and sieved to 0.5–1 mm fraction to avoid excessive pressure drop in the reactor. Z-sorb III is commercially available in pellets with 0.3 cm diameter and 0.6–0.9 cm long, with bulk density of 0.88 g/cc, but in order to test it in the same conditions, prior to the desulfurization experiments, Z-sorb III pellets were milled and sieved to obtain the 0.5–1 mm fraction. Graphene sorbents had a bulk density of approx. 1.1 g/cc. During the tests, no mechanical degradation was observed during the adsorption or regeneration tests. Briefly, 2.5 g of this fraction was placed in the reactor and the gas and water feeding systems were set to the desired values so that gas hourly space velocity was maintained at 3500 h^−1^. The reaction system was heated to the desulfurization temperature with a continuous flow of nitrogen through the reactor. When the desired temperature was reached, the nitrogen flow was stopped, and switched to the sulfidation atmosphere to start the experiment. Inlet and outlet gas composition were continuously determined by micro-GC and sulfidation progressed until a sharp increase in H_2_S concentration at the reactor outlet was noticed, which meant that the breakthrough point was achieved. At the end of each run, the used sorbent was discharged from the reactor, weighed and characterized.

#### 3.5.3. Regeneration Test

Oxidative regeneration of spent rGO-NiO-ZnO-400 was applied to bring the sorbent back to its original oxidation state. During regeneration, the following reactions took place (Equations (6) and (7)):


NiS + 3/2 O_2_ → NiO + SO_2_ (6)

ZnS + 3/2 O_2_ → ZnO + SO_2_ (7)

Regeneration conditions were selected in accordance with previous studies [33] and are shown in Table 7. 

Sorbent regeneration is maintained until SO_2_ concentration in the regeneration stream gas is not detected by microGC. At this point, the regeneration gas is switched to nitrogen to cool down the system. When the reactor reaches 400 °C, a new sulfidation cycle of the material begins again. The first set of experiments consisted of 3 sulfidations in nitrogen during 10 cycles while sulfidation in syngas streams were performed during 3 cycles.

## 4. Conclusions

We have prepared a NiO- and ZnO-doped rGO composite from graphite recovered by recycling spent Zn/C batteries. 

The oxidation and reduction processes of recycled graphite are able to remove impurities from the recycled graphite, resulting in high-quality and low-cost graphene-related materials fairly close to those synthesized from pure graphite. 

The desulfurization properties of the sorbent were investigated by exposing them to various operating conditions and atmospheres. Although there was a decrease in the desulfurization capacity compared to the sorbent prepared from pure graphite because a weaker reduction occurred, the proposed composite exhibited a capacity similar to the commercially available sorbents with fairly good response times. 

In conclusion, this work demonstrates that rGO-NiO-ZnO composites from recycled graphite have a great potential as a suitable and sustainable alternative to commercial desulfurization sorbents that encourages the development of more environmentally sustainable technologies for the industrial scale-up process. The proposed sorbent exhibited a superior performance at a low pressure which is clearly favorable, allowing its use without the need of gas compression upstream to the desulfurization reactor. Furthermore, the cycling tests showed that the rGO-NiO-ZnO sorbent shows acceptable stability with no drastic decay in the available capacity. Further studies should focus on the improvement of the desulfurization capacity and stability of the sorbents by exploring different experimental conditions for the reduction of GO during the hydrothermal and annealing treatments. 

## Figures and Tables

**Table 1:** Denomination of the materials involved in this work.

Denomination	Type of Material	Treatment
GRERV21-MX-SA	Recycled graphite	Recycled graphite from batteries treated with sulfuric acid
GO-17	Graphene oxide	Graphene oxide obtained using the Tour method and ultrasonic exfoliation
rGO-NiO-ZnO	Reduced graphene oxide functionalized with NiO and ZnO	Hydrothermally reduced graphene oxide, pre-functionalized with NiO and ZnO
rGO-NiO-ZnO-400	NiO and ZnO functionalized reduced graphene oxide with calcination at 400 °C.	Graphene oxide reduced by hydrothermal reduction, pre-functionalized with NiO and ZnO and subsequently subjected to 400 °C

**Table 2:** Results of the chemical characterization (%) carried out by WDXRF on the materials involved in this work. (-) means the absence of the element.

Element (%)	GRERV21-MX-SA	GO-17	rGO-NiO-ZnO	rGO-NiO-ZnO-400
C	63	39	8.5	3.9
Zn	0.080	-	37	47
Ni	-	-	5.0	6.2
Mn	9.6	0.31	-	-
Ba	1.3	1.2	0.16	0.22
Al	0.14	0.080	0.029	0.039
Br	0.037	0.020	-	-
Ca	0.0095	0.0041	-	-
Ce	-	-	0.07	0.12
Cl	0.94	0.31	-	0.022
Fe	0.46	0.020	0.011	0.016
K	0.15	0.15	-	-
La	0.054	0.032	-	-
P	0.017	0.055	0.0087	0.0089
Pb	0.041	0.043	-	-
S	0.66	1.8	0.18	0.26
Si	0.53	0.28	0.091	0.11
Ti	0.20	0.090	0.055	0.062

**Table 3:** The crystalline phases found in the synthesized materials.

Material	Compounds
GRERV21-MX-SA	C, BaSO_4_, SiO_2_
GO-17	BaK_x_SO_4_
rGO-NiO-ZnO	ZnO, Zn_4_(CO_3_)(OH)_6_H_2_O, Zn_5_(OH)_6_(CO_3_)_2,_ ZnSO_4_, 3Zn(OH)_2_
rGO-NiO-ZnO-400	ZnO, Ni_0.7_Zn_0.3_

**Table 4:** Experimental conditions.

N°	1	2	3
Reactor temperature (°C)	400	400	400
Pressure (bar)	1	1	1
Gas hourly space velocity GHSV (h^−1^)	3500	3500	3500
Sufidation gas composition (% *v*/*v*)			
H_2_S	0.9	0.3	0.3
N_2_	99.1	39.8	33.0
H_2_	-	26.1	22.7
CO	-	13.6	24.9
CO_2_	-	7.4	8.5
CH_4_	-	2.8	0.6
H_2_O	-	10	10

**Table 5:** Summary of experimental results and comparison of desulfurization performance.

N°	1	2	3
Theoretical Sulfur load capacity S_0_ (%)	26.4	26.4	26.4
Real Sulfur load capacity S_t_ (%)	24.1	27.1	22.8
Theoretical sorption time, t_0_ (min)	466	1389	1200
Breakthrough time (min)	414	1385	1008
Efficiency (S_t_/S_0_) (%)	91	102	86

**Table 6:** Summary of cyclability experimental results.

N° Test Type	1			2			3		
Cycle n°	1	2	3	1	2	3	1	2	3
Theoretical Sulfur load capacity S_0_ (%)	26.4			26.4			26.4		
Real Sulfur load capacity S_t_ (%)	24.1	23.7	22.8	27.1	24	24	22.8	23.1	23.7
Theoretical sorption time, t_0_ (min)	466			1389			1200		
Breakthrough time (min)	414	406	392	1385	1232	1230	1008	1021	1048
Efficiency (S_t_/S_0_) (%)	91	90	86	102	91	91	86	87	90

**Table 7:** Regeneration conditions.

Regeneration Conditions	
Reactor temperature (°C)	550
Pressure (bar)	1
Gas hourly space velocity GHSV (h^−1^)	2000
Regeneration gas composition (% *v*/*v*)	
N_2_	98
O_2_	2

## Data Availability

The original contributions presented in the study are included in the article, further inquiries can be directed to the corresponding author.

## References

[1] Government of Canada Graphite Facts. https://natural-resources.canada.ca/our-natural-resources/minerals-mining/mining-data-statistics-and-analysis/minerals-metals-facts/graphite-facts/24027.

[2] Statista, Mine Production of Graphite Worldwide from 2010 to 2023 (in 1000 Metric Tons). In Statista. https://www.statista.com/statistics/1005851/global-graphite-production/.

[3] Kara S., Hauschild M., Sutherland J., McAloone T. (2022). Closed-loop systems to circular economy: A pathway to environmental sustainability?. CIRP Ann..

[4] Chen W.-S., Liao C.-T., Lin K.-Y. (2017). Recovery Zinc and Manganese from Spent Battery Powder by Hydrometallurgical Route. Energy Procedia.

[5] Loudiki A., Mustapha M., Azriouil M., Laghrib F.-E., Farahi A., Bakasse M., Lahrich S., Mhammedi M. (2021). Graphene oxide synthesized from zinc-carbon battery waste using a new oxidation process assisted sonication: Electrochemical properties. Mater. Chem. Phys..

[6] Sperandio G., Machado Junior I., Bernardo E., Lopes R. (2023). Graphene Oxide from Graphite of Spent Batteries as Support of Nanocatalysts for Fuel Hydrogen Production. Processes.

[7] Vadivel S., Tejangkura W., Sawangphruk M. (2020). Graphite/Graphene Composites from the Recovered Spent Zn/Carbon Primary Cell for the High-Performance Anode of Lithium-Ion Batteries. ACS Omega.

[8] Chen I.W., Chen Y.-S., Kao N.-J., Wu C.-W., Zhang Y.-W., Li H.-T. (2015). Scalable and high-yield production of exfoliated graphene sheets in water and its application to an all-solid-state supercapacitor. Carbon.

[9] Kumar N., Salehiyan R., Chauke V., Joseph Botlhoko O., Setshedi K., Scriba M., Masukume M., Sinha Ray S. (2021). Top-down synthesis of graphene: A comprehensive review. FlatChem.

[10] Tian S., Sun J., Yang S., He P., Wang G., Di Z., Ding G., Xie X., Jiang M. (2016). Controllable Edge Oxidation and Bubbling Exfoliation Enable the Fabrication of High Quality Water Dispersible Graphene. Sci. Rep..

[11] Mbayachi V.B., Ndayiragije E., Sammani T., Taj S., Mbuta E.R., Khan A. (2021). Graphene synthesis, characterization and its applications: A review. Results Chem..

[12] Yam K.M., Guo N., Jiang Z., Li S., Zhang C. (2020). Graphene-Based Heterogeneous Catalysis: Role of Graphene. Catalysts.

[13] Fadlalla M., Ganesh Babu S. (2019). Role of graphene in photocatalytic water splitting for hydrogen production. Graphene-Based Nanotechnologies for Energy and Environmental Applications.

[14] Hasani A., Teklagne M., Do H., Hong S., Le Q., Ahn S.H., Kim S.Y. (2020). Graphene-based catalysts for electrochemical carbon dioxide reduction. Carbon Energy.

[15] Karimi S., Tavasoli A., Mortazavi Y., Karimi A. (2015). Enhancement of cobalt catalyst stability in Fischer–Tropsch synthesis using graphene nanosheets as catalyst support. Chem. Eng. Res. Des..

[16] Wang S., Sun H., Ang H.M., Tadé M.O. (2013). Adsorptive remediation of environmental pollutants using novel graphene-based nanomaterials. Chem. Eng. J..

[17] Yaengthip P., Siyasukh A., Payattikul L., Kiatsiriroat T., Punyawudho K. (2022). The ORR activity of nitrogen doped-reduced graphene oxide below decomposition temperature cooperated with cobalt prepared by strong electrostatic adsorption technique. J. Electroanal. Chem..

[18] Sánchez-Hervás J.M., Maroño M., Fernández-Martínez R., Ortiz I., Ortiz R., Gómez-Mancebo M.B. (2022). Novel ZnO-NiO-graphene-based sorbents for removal of hydrogen sulfide at intermediate temperature. Fuel.

[19] I.E.A. Bioenergy Task 33 Workshop: Biomass and Waste Gasification for the Production of Fuels..

[20] Sánchez-Hervás J., Ortiz I., Martí V., Andray A. (2023). Removal of Organic Sulfur Pollutants from Gasification Gases at Intermediate Temperature by Means of a Zinc–Nickel-Oxide Sorbent for Integration in Biofuel Production. Catalysts.

[21] Le P.A., Nguyen N.T., Nguyen P.L., Phung T.V.B. (2023). Minireview on Cathodic and Anodic Exfoliation for Recycling Spent Zinc-Carbon Batteries To Prepare Graphene Material: Advances and Outlook of Interesting Strategies. Energy Fuels.

[22] Thirumal V., Sreekanth T.V.M., Yoo K., Kim J. (2022). Facile Preparations of Electrochemically Exfoliated N-Doped Graphene Nanosheets from Spent Zn-Carbon Primary Batteries Recycled for Supercapacitors Using Natural Sea Water Electrolytes. Energies.

[23] Alcaraz L., Jiménez-Relinque E., Plaza L., García-Díaz I., Castellote M., López F.A. (2020). Photocatalytic Activity of Zn_x_Mn_3−x_O_4_ Oxides and ZnO Prepared From Spent Alkaline Batteries. Front. Chem..

[24] Al-Gaashani R., Najjar A., Zakaria Y., Mansour S., Atieh M.A. (2019). XPS and structural studies of high quality graphene oxide and reduced graphene oxide prepared by different chemical oxidation methods. Ceram. Int..

[25] Aliyev E.M., Khan M.M., Nabiyev A.M., Alosmanov R.M., Bunyad-zadeh I.A., Shishatskiy S., Filiz V. (2018). Covalently Modified Graphene Oxide and Polymer of Intrinsic Microporosity (PIM-1) in Mixed Matrix Thin-Film Composite Membranes. Nanoscale Res. Lett..

[26] Bayoumy A.M., Gomaa I., Elhaes H., Sleim M., Ibrahim M.A. (2021). Application of Graphene/Nickel Oxide Composite as a Humidity Sensor. Egypt. J. Chem..

[27] Gómez-Mancebo M.B., Fernández-Martínez R., Ruiz-Perona A., Rubio V., Bastante P., García-Pérez F., Borlaf F., Sánchez M., Hamada A., Velasco A. (2023). Comparison of Thermal and Laser-Reduced Graphene Oxide Production for Energy Storage Applications. Nanomaterials.

[28] Bukovska H., García-Perez F., Brea Núñez N., Bonales L.J., Velasco A., Clavero M.Á., Martínez J., Quejido A.J., Rucandio I., Gómez-Mancebo M.B. (2023). Evaluation and Optimization of Tour Method for Synthesis of Graphite Oxide with High Specific Surface Area. C.

[29] Kottegoda I.R.M., Idris N.H., Lu L., Wang J.-Z., Liu H.-K. (2011). Synthesis and characterization of graphene–nickel oxide nanostructures for fast charge–discharge application. Electrochim. Acta.

[30] Rabchinskii M.K., Sysoev V.V., Brzhezinskaya M., Solomatin M.A., Gabrelian V.S., Kirilenko D.A., Stolyarova D.Y., Saveliev S.D., Shvidchenko A.V., Cherviakova P.D. (2024). Rationalizing Graphene-ZnO Composites for Gas Sensing via Functionalization with Amines. Nanomaterials.

[31] Sánchez J.M., Ruiz E., Otero J. (2005). Selective Removal of Hydrogen Sulfide from Gaseous Streams Using a Zinc-Based Sorbent. Ind. Eng. Chem. Res..

[32] Das D., Das M., Sil S., Sahu P., Ray P.P. (2022). Effect of Higher Carrier Mobility of the Reduced Graphene Oxide–Zinc Telluride Nanocomposite on Efficient Charge Transfer Facility and the Photodecomposition of Rhodamine B. ACS Omega.

[33] Ozer L.Y., Garlisi C., Oladipo H., Pagliaro M., Sharief S.A., Yusuf A., Almheiri S., Palmisano G. (2017). Inorganic semiconductors-graphene composites in photo(electro)catalysis: Synthetic strategies, interaction mechanisms and applications. J. Photochem. Photobiol. C Photochem. Rev..

[34] Sánchez-Hervás J.M., Otero J., Ruiz E. (2005). A study on sulphidation and regeneration of Z-Sorb III sorbent for H_2_S removal from simulated ELCOGAS IGCC syngas. Chem. Eng. Sci..

[35] Frilund C., Simell P., Kaisalo N., Kurkela E., Koskinen-Soivi M.-L. (2020). Desulfurization of Biomass Syngas Using ZnO-Based Adsorbents: Long-Term Hydrogen Sulfide Breakthrough Experiments. Energy Fuels.

[36] Kawase M., Otaka M. (2013). Removal of H_2_S using molten carbonate at high temperature. Waste Manag..

[37] Rahim D.A., Fang W., Wibowo H., Hantoko D., Susanto H., Yoshikawa K., Zhong Y., Yan M. (2023). Review of high temperature H_2_S removal from syngas: Perspectives on downstream process integration. Chem. Eng. Process.-Process Intensif..

[38] Sasaoka E., Hirano S., Kasaoka S., Sakata Y. (1994). Characterization of reaction between zinc oxide and hydrogen sulfide. Energy Fuels.

[39] Selim H., Gupta A.K., Al Shoaibi A. (2012). Effect of CO_2_ and N_2_ concentration in acid gas stream on H_2_S combustion. Appl. Energy.

[40] Dhage P., Samokhvalov A., McKee M.L., Duin E.C., Tatarchuk B.J. (2013). Reactive adsorption of hydrogen sulfide by promoted sorbents Cu-ZnO/SiO2: Active sites by experiment and simulation. Surf. Interface Anal..

[41] Gil-Lalaguna N., Sánchez J.L., Murillo M.B., Gea G. (2015). Use of sewage sludge combustion ash and gasification ash for high-temperature desulphurization of different gas streams. Fuel.

[42] Lee J., Feng B. (2012). A thermodynamic study of the removal of HCl and H_2_S from syngas. Front. Chem. Sci. Eng..

[43] Thao Ngo T.N.L., Chiang K.-Y. (2023). Hydrogen sulfide removal from simulated synthesis gas using a hot gas cleaning system. J. Environ. Chem. Eng..

[44] Romo L.A., López-Fernández A., García-Díaz I., Fernández P., Urbieta A., López F.A. (2018). From spent alkaline batteries to ZnxMn_3_−xO_4_ by a hydrometallurgical route: Synthesis and characterization. RSC Adv..

[45] Marcano D.C., Kosynkin D.V., Berlin J.M., Sinitskii A., Sun Z.Z., Slesarev A., Alemany L.B., Lu W., Tour J.M. (2010). Improved Synthesis of Graphene Oxide. ACS Nano.

[46] Maroño M., Sánchez J.M., Ruiz E., Cabanillas A. (2008). Study of the Suitability of a Pt-Based Catalyst for the Upgrading of a Biomass Gasification Syngas Stream via the WGS Reaction. Catal. Lett..

